# Promoting Farmers’ Participation in Rural Settlement Environment Improvement Programmes: Evidence from China

**DOI:** 10.3390/ijerph19148585

**Published:** 2022-07-14

**Authors:** Dan Liu, Qianwen Gong

**Affiliations:** 1 School of Economics and Management, Beijing Forestry University, Beijing 100083, China; liu1123424@126.com; 2 School of Marxism, Beijing Forestry University, Beijing 100083, China

**Keywords:** farmer participation, rural settlement environment improvement, integrated weighting method, China

## Abstract

A rural settlement environment improvement programme is a livelihood project involving the vital interests of farmers. However, whether farmers should take the main responsibility for improving the rural settlement environment is an open issue. This study constructs an evaluation index system for farmers’ participation in rural settlement environment improvement on the basis of policy cognition, participation behaviour, and participation awareness. Using survey data from 909 farmers in eight provinces in China, this study empirically analyses farmers’ participation in a rural settlement environment improvement programme. The study’s results indicate that farmers have a high awareness of participation, a low level of policy cognition, and low involvement in the action regarding the rural settlement environment improvement. The participation of farmers in the rural settlement environment improvement is generally low and decreasing in the eastern, western, and central regions, in that order. Farmers’ participation in rural settlement environment improvement decreases in the order of suburban integration villages, characteristic protection villages, agglomeration and upgrading villages, and relocation and evacuation villages. To increase farmers’ participation in rural settlement environment improvement, the government can clarify the tasks in which farmers can participate, and establish an organisation and system to guide farmers’ involvement.

## 1. Introduction

The settlement environment is the geographical space where human production, life, and emotional communication occur. In the 1960s, the Greek urban planner Doxiadis proposed the concept of the ‘science of human settlement’. He stressed that the human settlement environment is a complex system of natural, social and artificial elements, there is a need for a science dealing with human settlements, because otherwise we cannot view these settlements in a reasonable way [1]. Early research on the human settlement environment focused on urban settlements. Scholars successfully evaluated the suitability of urban settlement environment, landscape change, spatial planning, construction form, environmental carrying capacity, and sustainable development [2,3,4,5,6,7]. At the same time, governments in developed countries have undertaken legislative and policy studies related to human settlements environmental protection. In 1969, the United States Congress approved the National Environmental Policy Act; hundreds of environmental protection and pollution control regulations have since been enacted that cover water, air, solid waste, noise, radiation, and other areas to form an entire system of environmental protection laws and regulations [8]. In the 1970s, Australia enacted the Victorian Environmental Protection Act, and Canada enacted the Canadian Water Pollution Control Act and the Water Act [9]. In 2000, the European Union issued the Water Framework Directive, which set a target of achieving a good status for all types of water in member states by 2015 [10]. A series of environmental protection efforts have been undertaken in various developed countries in relation to laws and regulations on environmental protection. Few studies in developed countries have focused specifically on rural settlement environment issues due to the narrow urban–rural divide. In developing countries, where the urban–rural divide is much larger, the environment of rural settlements is receiving increasing attention as the development of integrated urban and rural areas progresses.

In China, the concept of ‘rural’ is the direct opposite of ‘urban’. Rural areas are settlements formed by concentrations of agricultural production activities and an agricultural population, while urban areas are settlements formed by concentrations of nonagricultural production activities and a nonagricultural population. Rural settlements develop organically over time on the basis of farmers’ production activities and needs for accommodation, often relying on blood kinships and georelations. In 2021, the Law of the People’s Republic of China on the Promotion of Rural Revitalisation defined rural areas as a regional composite with natural, social, and economic characteristics and industrial, residential, ecological, cultural, and other functions, outside the built-up areas of a city, including any township, town, or village. Due to the limitations of the urban–rural dual system, China ignored the construction of rural human settlements in the early stages of development [11]. China has a large gap between rural and urban settlement environments for a long time. Rural settlement environment faces significant problems, including backward public service facilities, severe environmental pollution, and disorganised village constructions. The Secretary-General has repeatedly emphasised that national and regional development cannot be urban such as in Europe and rural such as in Africa, but should imply urban–rural regional coordination. Improving the rural settlement environment is crucial in China’s rural revitalisation strategy. The progress of the rural settlement environment affects the wellbeing of farmers, and the civilisation and harmony of rural society.

In 2018, the General Office of the Communist Party of China Central Committee and the General Office of the State Council put forward the Three-Year Action Programme for the Improvement of Rural Residential Environment. The main focus of the programme was on domestic waste treatment, domestic sewage treatment, toilet revolution and village appearance improvement. The programme’s objective was to achieve a marked improvement in the rural settlement environment by 2020 by making villages cleaner, tidier, and more orderly, and farmers more aware of their environment and health. All China’s provinces actively participated in the rural settlement environment improvement programmes. Since the implementation of the Three-Year Action Programme for the Improvement of Rural Residential Environment, the rural settlement environment has been improved. However, there is still a gap with the farmers’ aspiration for a better life. In 2021, the General Office of the Communist Party of China Central Committee and the General Office of the State Council proposed the Five-Year Action Plan for the Improvement of Rural Residential Environment. The programme’s objective is to achieve significant improvements in the rural settlement environment by 2025, and to achieve new progress in building ecologically liveable and beautiful villages. The government’s building activities have also exposed some problems. Farmers are not involved and are not satisfied with the results of the government’s construction, and allowing for farmers to play a leading role in the rural settlement environment improvement has become a focal issue. Hence, this study aims to promote farmers’ participation in the rural settlement environment improvement programmes.

There have been extensive discussions on farmers’ organisational forms and decision-making behaviours in the context of rural settlement environment improvement. Extant research on the form of farmers’ organisations mainly analyses the crucial roles played by nongovernmental organisations (NGOs) such as environmental cooperatives, agricultural committees, and community watershed organisations in educating and motivating farmers, and negotiating with the government [12,13,14]. Research on farmers’ decision making focuses on the determinants of decision making, such as the awareness of the environment and responsibility, the degree of access to information, neighbourhood relations, religious belief, economic and social status, institutional trust, local attachment, social norms, and social supervision [15,16,17,18,19,20,21,22,23,24]. It is not difficult to find that farmers being the subject in rural settlement environment improvement has globally become a social consensus [25,26,27,28]. However, whether farmers may take the lead in rural settlement environment improvement is an open issue. We designed an evaluation index system for farmers’ participation in rural settlement environment improvement on the basis of the theory of participatory governance. We used the proposed evaluation index system to measure farmers’ participation in rural settlement environment improvement in eight provinces in China: Gansu, Shaanxi, Anhui, Jiangsu, Sichuan, Yunnan, Heilongjiang, and Shanghai. In addition, we differentiate the measurement results by region and village type. The study’s results provide a reference for the systematic evaluation of farmers’ participation in rural settlement environment improvement and supports the formulation of suitable policies for promoting farmers’ involvement.

## 2. Materials and Methods

### 2.1. Research Design

On the basis of the theory of participatory governance, we constructed an evaluation index system for farmers’ participation in rural settlement environment improvement. Participatory governance is receiving increasing attention in the literature [29]. In recent years, developing countries have increasingly adopted participatory governance mechanisms to guide citizen participation in public policy formulation and implementation [30]. Empowerment [31], deliberative democracy [32], and a bottom–up approach [33] are the three main characteristics of participatory governance.

Empowerment means that participants can efficiently obtain public policy information. Deliberative democracy implies that participants can express their aspirations and demands. A bottom–up approach means that participants actively participate in formulating and implementing public policies. On the basis of participatory governance theory, Shen proposed that, in the context of the rural ecological environment, the characteristics of participatory governance are identification, empowerment, negotiation, and autonomy [28]. The author contends that ‘identity’ is expressed as farmers’ willingness to participate in governance when they understand rural environmental governance policies. ‘Empowerment’ is achieved by stimulating farmers’ consciousness, while ‘negotiation’ and ‘autonomy’ emphasise farmers’ collective action to participate in rural environmental governance.

Previous research has proven that policy cognition, participation awareness, and participation behaviour define ‘participation’. Hence, we constructed an evaluation index system of farmers’ participation in rural settlement environment improvement on the basis of these three aspects (Table 1). We measured policy cognition using farmers’ familiarity with policy information such as rural settlement environment improvement plans, tasks, and measures. We assessed participation awareness using farmers’ consciousness of the subjects, recognition, and initiatives in rural settlement environment improvement programmes. Lastly, we measured participation behaviour using farmers’ participation in formulating, implementing, and modifying rural settlement environment improvement plans.

### 2.2. Questionnaire Design

Table 2 reports the questionnaire’s items corresponding to each indicator’s layer. We rated the answers using a five-level Likert scale. Farmers’ participation in rural settlement environment improvement ranged from 1 to 5, indicating increasing involvement.

### 2.3. Data Collection

#### 2.3.1. Research Areas

**Figure 1:**
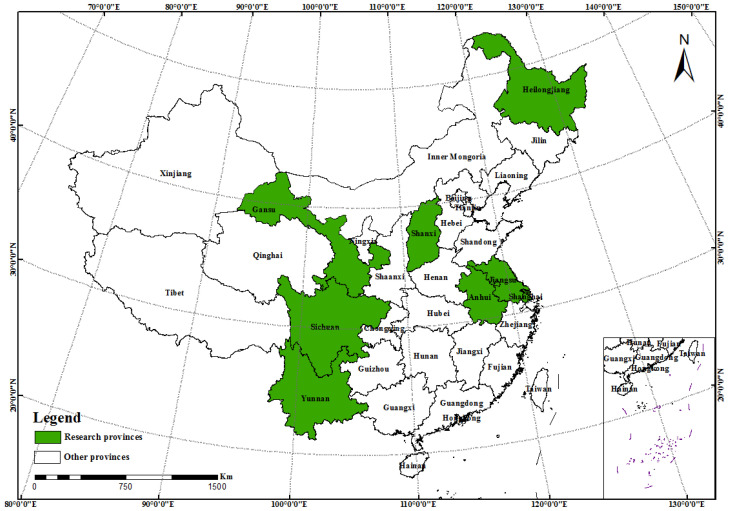
Distribution of research provinces.

This study addresses eight provinces in China, namely, Gansu, Shaanxi, Anhui, Jiangsu, Sichuan, Yunnan, Heilongjiang, and Shanghai. The specific distribution of each province is shown in Figure 1. From July to September 2019, the research group organised three PhD students and nine MSc students, and conducted a survey in the above-mentioned provinces. We selected different villages in the same province for investigation because a village’s economic and social development directly impacts the progress and farmers’ participation in rural settlement environment improvement (Table 3). In line with the Strategic Plan for Rural Vitalisation (2018–2022), we chose suburban integration villages, characteristic protection villages, agglomeration and upgrading villages, and relocation and evacuation villages. There are differences in the location conditions and resource endowments of the four types of villages. Suburban integration villages are either villages close to cities or villages where township governments are located. Characteristic protection villages are villages with historical and cultural resources, and rural tourism resources. Agglomeration and upgrading villages are central villages with a large populations and stable economic development. Relocation and evacuation villages are villages in areas characterised by poor living conditions and a fragile ecological environment, and are prone to frequent natural disasters. The division of the surveyed villages by type allows for analysing the similarities and differences in farmers’ participation in rural settlement environment improvement at a national level.

All the members of the survey team were from rural areas, and have a good understanding of rural living conditions and lifestyles, agricultural production conditions, and the behavioural characteristics of farmers. Most of the members of the survey team also had experience in conducting rural surveys. The survey took the form of one-to-one question-and-answer sessions. To further ensure the accuracy of the survey results, all the survey team members received professional training before the formal survey. In addition, three farmers were selected by each survey team member for a pilot survey. The questionnaire was modified on the basis of the results of the pilot survey to ensure that the farmers in the official survey would be able to fully understand what was being asked. A total of 909 samples were collected.

#### 2.3.2. Sample Characteristics

The specific statistical results are shown in Table 4. There were 650 male respondents (71.51%) and 259 female respondents (28.49%). The gender difference can be explained by the fact that male farmers are generally the household decision makers. Most of the respondents fell into the following age groups: 45–55 (30.03%), 55–65 (26.29%), and over 65 (30.14%). The low proportion of respondents under the age of 45 was due to the fact that most young and middle-aged people in rural areas go out to work. In total, 12.32% of the respondents were illiterate. Most of the respondents (73.27%) only had a junior or senior middle-school education. In terms of the respondents’ family structure, the number of respondents’ family members grouped as less than or equal to 3 and 3 to 5 accounted for the main proportion (79.42%), with the number of family members engaged in nonagricultural activities mainly less than or equal to 2. Nonagricultural income was an important source of income for many families. The annual household income of the respondents was characterised by a clear polarisation, with 27.94% and 21.45% of the respondents having an annual household income of less than RMB 20,000 and more than RMB 80,000, respectively.

### 2.4. Mathematical Model

An integrated weighting method was constructed to calculate the weights of the evaluation indicators of farmers’ participation in rural settlement environment improvement. The proposed method achieves an organic combination of subjective and objective weighting methods. It reflects the experts’ understanding of the research problem using acquired knowledge and research experience. It also considers rigorous mathematical theory and methods for the scientific calculation of indicators values.

First, the analytic hierarchy process (AHP) was used to calculate the evaluation indicators’ weights $w_{j}^{\prime }$. Second, the entropy weighting method (EWM) was employed to assess evaluation indicators’ weights $w_{j}^{\prime \prime }$. Lastly, the evaluation indicators’ weights w were calculated on the basis of both approaches.

#### 2.4.1. AHP

AHP is a method to calculate an indicator’s weight by layering the evaluation-related elements and constructing a comparison matrix. It calculates indicators weights through five steps:

*Step* 1: establishing a hierarchical structure model;

*Step* 2: constructing a two-by-two comparison judgement matrix:

(1) $$A={\left(a_{jj}\right)}_{n\times n}=\left[\begin{array}{cccc} a_{11} & a_{12} & \Lambda & a_{1n}\\ a_{21} & a_{22} & \Lambda & a_{2n}\\ \Lambda & \Lambda & \Lambda & \Lambda \\ a_{n1} & a_{n2} & \Lambda & a_{nn} \end{array}\right]$$

where $a_{ij}$ is the importance of $a_{i}$ relative to $a_{j}$, $a_{ij}>0$, $a_{ii}=1$, and $a_{ij}=\frac{1}{a_{ji}}$;

*Step* 3: Calculating the relative weights of indicators under a single criterion. The relative weight of the *j*th indicators is calculated using the characteristic root method and normalised to obtain the weight of the corresponding indicators in a given level compared to an indicator in the previous level. The calculation formula reads as follows:

(2) $$w_{j}^{\prime }=n\sqrt{\overset{n}{\underset{i=1}{\prod }}a_{ij}}/\overset{n}{\underset{j=1}{\sum }}\left(n\sqrt{\overset{n}{\underset{i=1}{\prod }}a_{ij}}\right)$$

The maximal eigenvalue of the judgment matrix is $\lambda_{max}$:

(3) $$\lambda_{max}=\overset{n}{\underset{j=1}{\sum }}\frac{{\left(AW\right)}_{i}}{{nW}_{i}}$$

where ${\left(AW\right)}_{I}$ denotes the *i*th element of the vector *AW.*

*Step* 4: Consistency check for hierarchical single ordering. The closer the CI of the consistency indicator is to zero, the more satisfactory the consistency result:

(4) $$CI=\frac{\lambda_{max}-n}{n-1}$$

*Step* 5: Consistency check for hierarchical total ordering. A judgement matrix is considered to be satisfactorily consistent if *CR* is less than or equal to 0.1:

(5) $$CR=\frac{CI}{RI}$$

RI is the average random consistency index.

#### 2.4.2. EWM

EWM evaluates a value by measuring its degree of differentiation. The greater the degree of dispersion is, the greater the degree of differentiation is, and the more information can be derived. Hence, a higher weight should be given to the indicator [34]. In this method, $X_{ij}\;(i=1,\;2,\;\ldots ,\;m;\;j=1,\;2,\;\ldots ,\;n)$ is the value of the *j-*th evaluation indicators in the *i-*th sample. EWM calculates indicators’ weights through four steps:

*Step* 1: Standardising measured values. The standardised value of the *j-*th indicators in the *i-*th sample is denoted as $p_{ij}$; it is calculated as follows:

(6) $$p_{ij}{=X}_{ij}/\overset{m}{\underset{i=1}{\sum }}X_{ij}$$

*Step* 2: calculating the entropy value $e_{j}\;$ of the *j-*th indicators:

(7) $$e_{j}=-\left(1/lnm\right)\overset{m}{\underset{i=1}{\sum }}p_{ij}{lnp}_{ij}$$

*Step* 3: calculating coefficient of variation $g_{j}$ for the *j-*th indicators:

(8) $$g_{j}=1-e_{j}$$

The lower the entropy value is, the greater the differentiation degree of indicators, and the more important the indicators are.

*Step* 4: Defining evaluation indicators weights $w_{j}^{\prime \prime }$:

(9) $$w_{j}^{\prime \prime }=g_{j}/\overset{n}{\underset{j=1}{\sum }}g_{j}$$

#### 2.4.3. Integrated Weighting Method (IWM)

On the basis of the indicator’s weights calculated by the AHP and EWM, the final evaluation indicators weights are as follows:

(10) $$W=\alpha w^{\prime }+\beta w^{\prime \prime }$$

where $w=(w_{1}^{\prime },w_{2}^{\prime },\;\ldots ,w_{n}^{\prime })$, $0\leq w_{j}^{\prime }\leq 1,$ and $\sum_{j=1}^{n}w_{j}^{\prime }=1$. In addition, $w^{\prime \prime }=(w_{1}^{\prime \prime },w_{2}^{\prime \prime },\;\ldots ,w_{n}^{\prime \prime })$, $0\leq w_{j}^{\prime \prime }\leq 1,$ and $\sum_{j=1}^{n}w_{j}^{\prime \prime }=1$; α and β denote the importance of $w^{\prime }$ and $w^{\prime \prime }$, respectively.

If α and β satisfy the unitisation constraint conditions,

(11) $$\alpha^{2}+\beta^{2}=1$$

According to the weighting rule of multiattribute decision analysis, the evaluation target value of each evaluation object can be calculated as follows:

(12) $$d_{i}=\overset{n}{\underset{j=1}{\sum }}X_{ij}w_{j}=\overset{n}{\underset{j=1}{\sum }}X_{ij}(\alpha w_{j}^{\prime }+\beta w_{j}^{\prime \prime })\;(i=1,2,\ldots ,m)$$

where $X_{ij}$ is the value of each evaluation indicator. In general, the larger the $d_{i}$, the better. Therefore, the construction of a multiobjective planning model implies:

(13) $$max\;D=(d_{1},d_{2}\ldots ,\;d_{m})$$

A multiobjective planning model can be synthesised by the linear weighted sum method into an equivalent single-objective optimisation model, as follows:

(14) $$max\;Z=\overset{m}{\underset{i=1}{\sum }}d_{i}=\overset{m}{\underset{i=1}{\sum }}\overset{n}{\underset{j=1}{\sum }}X_{ij}{(\alpha w}_{j}^{\prime }{+\beta w}_{j}^{\prime \prime })$$

Equations (13) and (14) satisfy the constraint conditions: $\alpha^{2}+\beta^{2}=1,\;\mathrm{and}\;\alpha ,\;\beta \geq 0$. The single-objective optimisation model can be solved by constructing a Lagrange function. By normalizing the solution results, the optimal solutions $\alpha^{*},\;\beta^{*}$ of the optimization model can be obtained as follows:

(15) $${\bar{\alpha }}^{*}=\overset{m}{\underset{i=1}{\sum }}\overset{n}{\underset{j=1}{\sum }}X_{ij}w_{j}^{\prime }/\overset{m}{\underset{i=1}{\sum }}\overset{n}{\underset{j=1}{\sum }}X_{ij}\left(w_{j}^{\prime }{+w}_{j}^{\prime \prime }\right)$$

(16) $${\bar{\beta }}^{*}=\overset{m}{\underset{i=1}{\sum }}\overset{n}{\underset{j=1}{\sum }}X_{ij}w_{j}^{\prime \prime }/\overset{m}{\underset{i=1}{\sum }}\overset{n}{\underset{j=1}{\sum }}X_{ij}\left(w_{j}^{\prime }{+w}_{j}^{\prime \prime }\right)$$

#### 2.4.4. Participation Calculation Model

Combining the above analysis, and taking ${\bar{\alpha }}^{*}$ and ${\bar{\beta }}^{*}$ as the coefficients of objective and subjective weights, the model for measuring farmers’ participation in rural habitat improvement reads as follows:

(17) $${\bar{d}}_{i}=\overset{n}{\underset{j=1}{\sum }}X_{ij}\left({\bar{\alpha }}^{*}w_{j}^{\prime }+{\bar{\beta }}^{*}w_{j}^{\prime \prime }\right)$$

## 3. Results

### 3.1. Indicators’ Weights

The results of Equations (15) and (16) indicate that ${\bar{\alpha }}^{*}$ and ${\bar{\beta }}^{*}$ were 0.5609 and 0.4391, respectively. Table 5 reports the weights of each evaluation indicator. Significant differences were observed in the weights measured by the AHP and the EWM. These differences are mainly in the weighting of the first-level indicators.

The results of the AHP method indicate that the weights of participation behaviour and participation awareness were 55.56% and 33.33%, respectively. This result supports the view that farmers’ participation in rural settlement environment improvement focuses on practical actions and identification with environmental improvement. In contrast, the importance of understanding rural human settlement environment improvement policies is modest.

Among the indicators weights calculated by EWM, little difference was observed in the weights of the three first-level indicators of policy cognition, participation awareness, and participation behaviour. This result indicates no significant differences in respondents’ participation in rural settlement environment improvement across the three first-level indicators. Overall, the ranking of the weights of the secondary indicators measured by the AHP and EWM was consistent

### 3.2. Analysis

This study provides a comparative analysis of farmers’ involvement from multiple perspectives. First, we compared the involvement of farmers in each research province. Then, we compared the participation of farmers in the eastern, central, and western regions. Lastly, we compared the participation of farmers in different types of villages. The highest participation index of farmers was 5, in which the highest policy cognition index was 1.02, the highest participation awareness index was 1.70, and the highest participation behaviour index was 2.28.

#### 3.2.1. Farmers’ Participation in the Research Provinces

The results in Table 6 indicate that farmers’ participation in rural settlement environment improvement is generally low in the research provinces, with an average participation index of 3.1387. Yunnan had the highest participation index at 3.5723, followed by Shanghai and Jiangsu Province with 3.4709 and 3.1959, respectively, while Gansu had the lowest participation index at 2.8787. Two reasons may explain these results. First, the policy was not designed assuming farmers as the main participants. When local governments undertake the improvement of the rural settlement environment, they directly outsource the project, and rural collective economic organisations and farmers passively accept it with reduced possibilities of active participation. Second, farmers’ demand for rural settlement environment improvement is low, as they experience insufficient incentives for active participation. Farmers do not generally believe that environmental improvement is meaningful. Shanxi had the lowest participation index of policy cognition for farmers at 0.4364, 0.1756 lower than the qualified level. This result indicates insufficient publicity for the policy supporting rural settlement environment improvement in Shanxi. Yunnan had the highest participation index of participation awareness. This finding reflects Yunnan’s attention to ecological environment protection and the development of the ecological economy. The participation index in terms of participation behaviour varies considerably, with a difference of 0.4698 between Yunnan, which had the highest participation index, and Gansu, which had the lowest.

#### 3.2.2. Farmers’ Participation by Region

Previous studies have shown that the level of economic and social development is a crucial determinant of farmers’ participation in rural settlement environment improvement. Therefore, this research analyses the difference in farmers’ participation in regional rural settlement environment improvement. Table 7 shows that the involvement of farmers in rural settlement environment improvement in the research provinces is decreasing in the eastern, western, and central regions, in that order. First, the participation index of the policy cognition of farmers in the central region is 0.1133 lower than that of farmers in the western region. The western region has a fragile ecological environment compared to the central region. The local government places great emphasis on protecting the ecological environment, constantly increasing its efforts to promote environmental improvement policies. Second, farmers in the central region had a lower index of participation behaviour than those in the eastern region by 0.1483. This result is mainly due to the relatively high level of economic development of villages in the eastern region, where farmers have already completed the ‘toilet revolution’ and developed the habit of disposing of domestic waste and sewage. However, some villages in the central region are still in the initial stage of settlement environment improvement, and some farmers have low demand for environmental improvement and low willingness to participate. Lastly, the difference in farmers’ index of participation awareness among the eastern, central, and western regions’ research provinces is relatively small. Overall, farmers have a high degree of recognition for improving the rural settlement environment.

#### 3.2.3. Farmers’ Participation by Village Type

Table 8 reports the results of farmers’ participation in improving the rural settlement environment in different types of villages. The results show that suburban integration villages, and the relocation and evacuation villages exhibited the highest and lowest farmers’ participation index, respectively. At the same time, the participation indices of farmers’ policy awareness and participation behaviour in the relocation and evacuation villages are much lower than those in the other three types of villages. In particular, the participation index of farmers’ participation behaviour was 0.5163 lower in relocation and eviction villages than that in suburban integration villages. The difference in location conditions between suburban integration villages, and relocation and eviction villages significantly affects farmers’ participation in improving the rural settlement environment. Suburban integration villages have unique geographical advantages, enjoy the economic and cultural spillover generated by urban development, and farmers’ awareness and participation behaviour are relatively high. However, the relocation and eviction of villages are constrained by ecological and environmental conditions, with insufficient internal motivation for development, and strong dependence on external forces; besides, farmers’ consciousness has not been fully formed. In addition, characteristic protection villages exhibit the highest index of participation in policy awareness at 0.6426. The participation index of characteristic protection villages is much higher than that of agglomeration and upgrading villages. Characteristic protection villages have profound cultural heritage and great potential for developing rural tourism and characteristic industries; hence, farmers are motivated to participate in rural settlement environment improvement. In contrast, agglomeration and upgrading villages are generally larger; farmers are household-based, lacking a solid action structure and showing weaker action in rural settlement environment improvement.

## 4. Discussion and Conclusions

### 4.1. Discussion

This study empirically analyses farmers’ participation in rural settlement environment improvement in China by addressing provinces, regions, and villages. The study’s main conclusions are as follows.

First, farmers’ participation in rural settlement environment improvement is generally low in the research provinces. Although farmers agree with the rural settlement environment improvement policy and are willing to maintain the rural settlement environment, they are unwilling to invest money or labour to improve it. Second, the participation of farmers in the improvement of the rural settlement environment shows a decreasing trend in the eastern, western, and central regions, in that order. The participation index of farmers’ participation behaviour in the central region is much lower than that of farmers in the east and west. Third, farmers’ participation in rural settlement environment improvement decreases in the order of suburban integration villages, characteristic protection villages, agglomeration and upgrading villages, and relocation and evacuation villages. Farmers in suburban integrated villages have higher participation indices in policy cognition, participation awareness, and participation behaviour than those of the other three types of villages.

Despite its contributions, this research has some limitations that suggest a few future research directions. First, this study only theoretically analyses the reasons for the low participation of farmers in rural settlement environment improvement and the spatial differences in participation. Future research should analyse the key factors affecting farmers’ participation in rural settlement environment improvement and the spatial heterogeneity of the influencing factors. Second, the research conclusions are based on the survey data of 909 farmers in China. Future studies should expand the sample size to further analyse the provincial differences in farmers’ participation in rural settlement environment improvement in the same type of villages.

### 4.2. Conclusions

Farmers’ participation in rural settlement environment improvement is the key to ensuring the sustainable promotion of rural settlement environment improvement programmes. To improve their participation in the programme, we propose an evaluation index system to assess farmers’ participation on the basis of policy cognition, participation behaviour, and participation awareness in line with participatory governance theory.

The research results have practical significance for policies aimed at enhancing farmers’ participation in rural settlement environment improvement, identifying the shortcomings of farmers’ involvement, and providing a basis for constructing other citizens’ participation evaluation index systems. Overall, the study’s results indicate that farmers play a marginal role in improving the rural settlement environment; significant room exists to enhance participation, potentially impacting rural settlement environmental improvement.

On the basis of the above conclusions, this research mainly has the following policy implications. First, local governments in China are essential innovative agents of institutional reform. Local government management innovation is vital in guiding and promoting collaborative social development. Therefore, local governments can innovatively formulate rural settlement environment improvement programmes with farmers as the main actors in policy practice, guiding them to take responsibility for implementing some of the improvement tasks and maintaining the improvement achievements. Second, local governments are encouraged to formulate policies to guide elite farmers to set up rural settlement environment improvement associations. As a social organisation, the association can be close to the grassroots, allowing for farmers to participate in rural settlement environment improvement in an organised manner. It can also establish a consultation and communication mechanism with the local government, effectively dealing with rural settlement environment improvement matters. In order to increase the motivation of local governments to promote the establishment of rural settlement environment improvement associations, we can use the development of rural environmental protection social organisations as an essential indicator in the performance evaluation of rural settlement environment improvement by local governments. We can also establish a targeted financial subsidy mechanism for local governments to purchase the services of rural settlement environment improvement associations. Third, the responsibilities of farmers in rural settlement environment improvement should be clarified by formulating village development plans and identifying the village development types.

This study explored the evaluation index system of farmers’ participation in rural settlement environment improvement and discusses the actual situation of farmers’ participation in rural settlement environment improvement in China. This study enriches the application of participatory governance theory in the field of rural settlement environment improvement. It lays the foundation for evaluating farmers’ participation in rural settlement environment improvement, which helps the government in motivating farmers to participate in rural settlement environment improvement and build a better rural settlement environment.

## Figures and Tables

**Table 1:** Evaluation index system of farmers’ participation in rural settlement environment improvement.

Target Layer (A)	Element Layer (B)	Indicator Layer (C)
Farmers’ participation in rural settlement environment improvement	Policy cognition (B1)	Farmers’ familiarity with environment improvement programmes (C1).
		Farmers’ familiarity with environment improvement tasks (C2).
		Farmers’ familiarity with environment improvement measures (C3).
	Participation awareness (B2)	Subject consciousness (C4).
		The necessity of farmers’ participation in environmental improvement (C5).
		Attitude towards punishment for damaging the environment (C6).
		Recognition of rural settlement environmental improvement (C7).
	Participation behaviour (B3)	Participation in the formulation of improvement programmes (C8).
		Participation in improving the appearance of the village (C9).
		Participation in the renovation of toilets (C10).
		Participation in the disposal of domestic waste (C11).
		Participation in the treatment of domestic sewage (C12).
		Proactively monitoring others in the village to protect the environment (C13).
		Participation in policy or knowledge dissemination activities related to environment improvement (C14).
		Making policy recommendations to the rural peasant collective regarding environmental improvement (C15).
		Proactive protection of the environment in public areas (C16).

**Table 2:** Measurement of participation.

Indicator Layer	Question	Assignment
C1	Do you know the ‘three-year action plan for improving rural settlement’?	I do not know at all = 1; I know a little = 2; general knowledge = 3; I know roughly = 4; I know very clearly = 5.
C2	Do you know the critical tasks of rural settlement environment improvement?	I know less than or one task = 1; I know two tasks = 2; I know three tasks = 3; I know four tasks = 4; I know five or more than five tasks = 5.
C3	Do you know the specific measures of the ‘toilet revolution’?	I do not know at all = 1; I know a little = 2; general knowledge = 3; I know roughly = 4; I know very clearly = 5.
C4	Who should be primarily responsible for rural settlement environment improvement?	Government = 1; rural collective economic organisation = 2; combination of farmers and government = 3; combination of farmers and rural collective economic organisation = 4; farmers = 5.
C5	Do you think it is necessary to organise farmers to improve the rural settlement environment?	Totally unnecessary = 1; hardly necessary = 2; generally necessary = 3; a little necessary = 4; very necessary = 5.
C6	Do you think punishing people who destroy the rural settlement environment is necessary?	Totally unnecessary = 1; hardly necessary = 2; generally necessary = 3; a little necessary = 4; very necessary = 5.
C7	Do you agree with the idea that ‘clear waters and lush mountains are invaluable assets’?	Totally disagree = 1; hardly agree = 2; generally agree = 3; partly agree = 4; totally agree = 5.
C8	Have you ever participated in formulating the rural settlement environment improvement plan for your village?	I never participated = 1; I participated in less than half = 2; I participated in half = 3; I participated in more than half = 4; I participated in all = 5.
C9	Would you like to invest some money or labour to change the appearance of your village?	Totally unwilling = 1; almost unwilling = 2; generally unwilling = 3; partly willing = 4; very willing = 5.
C10	If your village has carried out toilet reconstruction, have you participated?	I never participated = 1; I participated in less than half = 2; I participated in half = 3; I participated in more than half = 4; I participated in all = 5.
	If your village has not carried out toilet reconstruction, would you be willing to pay for installing a toilet?	Totally unwilling = 1; almost unwilling = 2; generally unwilling = 3; partly willing = 4; very willing = 5.
C11	If your village has standardised household waste disposal, are you now used to disposing of your household waste at a designated place?	Not used to it at all = 1; a little unused to it = 2; generally not used to it = 3; a little used to it = 4; very used to it = 5.
	If your village has no standardised disposal of household waste, would you like to invest some money or labour in the disposal of the village’s household waste?	Totally unwilling = 1; almost unwilling = 2; generally unwilling = 3; partly willing = 4; very willing = 5.
C12	If your village has standardised domestic sewage treatment, are you now used to dumping it in a designated place?	Not used to it at all = 1; a little unused to it = 2; generally not used to it = 3; a little used to it = 4; very used to it = 5.
	If your village has no standardised domestic sewage treatment, would you like to invest some money or labour to construct a sewage treatment plant?	totally unwilling = 1; almost unwilling = 2; general = 3; partly willing = 4; very willing = 5.
C13	Do you proactively supervise others to protect the rural settlement environment?	No supervision = 1; very little supervision = 2; general = 3; frequent supervision = 4; always supervise = 5.
C14	Have you ever participated in policy or knowledge dissemination activities for environmental improvement?	I never participated = 1; I participated once = 2; I participated twice = 3; I participated three times = 4; I participated more than three times = 5.
C15	Have you made policy recommendations to the rural peasant collective on environmental improvement?	I never make suggestions = 1; I hardly make suggestions = 2; I generally make suggestions = 3; I often make suggestions = 4; I always make suggestions = 5.
C16	Do you take the initiative to clean the public areas of the village?	I never clean up = 1; I hardly clean = 2; I generally clean = 3; I often clean = 4; I always clean = 5.

**Table 3:** Research area and distribution of sample farmers.

Research Province	Research City	Research Village	Village Type	Sample Size
Gansu	Baiyin	Huapichuan	Agglomeration and upgrading	62
	Dingxi	Dacha	Relocation and evacuation	28
	Tianshui	Shiziping	Suburban integration	62
Shanxi	Jinzhong	Pangzhuang	Relocation and evacuation	46
	Jincheng	Dazhang	Suburban integration	63
Jiangsu	Huaian	Gaoqiao	Characteristic protection	61
	Zhenjiang	Xifeng	Agglomeration and upgrading	60
Anhui	Huangshan	Jianfeng	Agglomeration and upgrading	62
	Chuzhou	Ranzhou	Agglomeration and upgrading	62
Shanghai	Pudong	Jiebang	Agglomeration and upgrading	64
Heilongjiang	Daqing	Donggangzi	Relocation and evacuation	61
	Haerbin	Xinyi	Suburban integration	62
Sichuan	Neijiag	Majiasi	Characteristic protection	58
	Chengdu	Anlong	Suburban integration	40
Yunnan	Lincang	Anshi	Suburban integration	62
	Dali	Jiangdeng	Characteristic protection	56

**Table 4:** Sample characteristics.

Sample (%)		All	Gansu	Anhui	Jiangsu	Shanxi	Shanghai	Heilongjiang	Sichuan	Yunnan
Gender	Male	71.51	75.66	37.90	84.30	51.38	78.13	75.61	68.37	76.27
	Female	28.49	24.34	62.10	15.70	48.62	21.88	24.39	31.63	23.73
Age	≤35	4.73	5.26	6.45	0.00	6.41	0.00	4.07	5.10	8.47
	35~45	8.81	7.89	5.65	4.13	15.60	1.56	12.20	7.14	13.56
	45~55	30.03	31.58	33.06	18.18	32.11	14.06	26.83	40.82	38.14
	55~65	26.29	29.61	19.36	23.14	26.61	32.82	33.32	18.37	27.97
	>65	30.14	25.66	35.48	54.55	19.27	51.56	23.58	28.57	11.86
Degree of education	Illiteracy	12.32	9.87	33.06	23.97	8.26	1.56	3.25	12.24	0.85
	Primary school	32.56	31.58	20.16	35.53	22.02	18.75	47.51	39.80	39.83
	Junior middle school	40.71	32.89	38.72	28.10	58.72	57.81	38.22	39.80	43.22
	Senior middle school	10.67	21.05	8.06	6.61	8.26	17.19	6.50	8.16	9.32
	Junior college	3.19	3.95	0.00	5.79	2.74	3.13	4.88	0.00	4.24
	Undergraduate or above	0.55	0.66	0.00	0.00	0.00	1.56	0.00	0.00	2.54
Number of family members	≤3	40.37	33.55	35.48	44.63	58.72	35.94	68.29	34.69	11.02
	3~5	39.05	40.13	41.13	38.02	32.11	39.06	26.02	43.88	52.54
	5~7	17.93	22.37	18.55	15.70	9.17	23.44	3.25	20.41	32.20
	>7	2.65	3.95	4.84	1.65	0.00	1.56	2.44	1.02	4.24
Number of family members engaged in nonagriculture	0	26.95	23.68	22.58	36.36	28.44	25.00	46.34	17.35	13.56
	1	20.57	20.39	19.35	13.22	30.28	15.63	16.26	29.59	20.34
	2	31.35	30.93	34.68	28.10	26.60	20.31	22.76	40.82	43.22
	3	12.65	11.84	11.29	14.88	11.93	32.81	8.13	6.12	12.71
	>3	8.48	13.16	12.10	7.44	2.75	6.25	6.51	6.12	10.17
Annual income of the family (RMB)	≤20,000	27.94	36.84	37.10	19.01	40.37	0.00	39.02	22.45	12.72
	20,000~40,000	25.74	32.89	26.61	16.53	38.53	1.56	17.89	31.63	29.66
	40,000~60,000	16.94	16.45	20.16	19.00	14.68	3.13	14.63	23.47	18.64
	60,000~80,000	7.93	6.58	4.84	12.40	3.67	14.06	7.32	7.14	10.17
	>80,000	21.45	7.24	11.29	33.06	2.75	81.25	21.14	15.31	28.81

**Table 5:** Weights of the evaluation indicators of farmers’ participation.

First-Level Indicators	Weight			Secondary Indicators	Weight		
	Analytic Hierarchy Process	Entropy Weight Method	Integrated Weighting Method		Analytic Hierarchy Process	Entropy Weight Method	Integrated Weighting Method
Policy cognition B1	0.1111	0.3246	0.2048	C1	0.0185	0.1684	0.0843
				C2	0.0370	0.0928	0.0615
				C3	0.0556	0.0634	0.0590
Participation awareness B2	0.3333	0.3488	0.3401	C4	0.1135	0.2482	0.1726
				C5	0.1025	0.0835	0.0942
				C6	0.0587	0.0108	0.0377
				C7	0.0587	0.0062	0.0356
Participation behaviour B3	0.5556	0.3267	0.4550	C8	0.0299	0.0823	0.0529
				C9	0.0773	0.0204	0.0523
				C10	0.0897	0.0512	0.0728
				C11	0.0897	0.0146	0.0567
				C12	0.0897	0.0110	0.0551
				C13	0.0299	0.0255	0.0280
				C14	0.0299	0.0683	0.0468
				C15	0.0598	0.0395	0.0509
				C16	0.0598	0.0139	0.0396

**Table 6:** Farmers’ participation index in rural settlement environment improvement.

Province	Farmers’ Participation Index			
	Policy Cognition	Participation Awareness	Participation Behaviour	Total
Gansu	0.5037	1.1462	1.2287	2.8787
Anhui	0.4927	1.1117	1.3660	2.9704
Jiangsu	0.6058	1.1239	1.4662	3.1959
Shanxi	0.4364	1.1735	1.4229	3.0328
Shanghai	0.6411	1.1403	1.6895	3.4709
Heilongjiang	0.5433	1.0781	1.3851	3.0065
Sichuan	0.6481	0.9147	1.4189	2.9817
Yunnan	0.6477	1.2260	1.6985	3.5723

**Table 7:** Farmers’ participation index in rural settlement environment improvement by region.

Region	Farmers’ Participation Index			
	Policy Cognition	Participation Awareness	Participation Behaviour	Total
East	0.5883	1.3392	1.5259	3.4534
Central	0.5121	1.3310	1.3809	3.2239
West	0.6254	1.3368	1.4722	3.4344

**Table 8:** Farmers’ participation index in rural settlement environment improvement by village type.

Village Type	Farmers’ Participation Index			
	Policy Cognition	Participation Awareness	Participation Behaviour	Total
Agglomeration and upgrading	0.5370	1.3379	1.4178	3.2927
Characteristic protection	0.6426	1.2846	1.4576	3.3848
Relocation and evacuation	0.4855	1.3030	1.1397	2.9283
Suburban integration	0.6082	1.3885	1.6560	3.6527

## Data Availability

The raw data supporting the conclusions of this article are made available by the authors without undue reservation to any qualified researcher.
